# Patients' Payments in District Work

**Published:** 1909-09-18

**Authors:** 


### Nursing Administration.

### PATIENTS' PAYMENTS IN DISTRICT WORK.

As district work expands and takes root in one fresh neighbourhood after another, the question of payment or contributions from patients becomes very important. Is the boon of nursing in sickness to be one which the rich bestow on their poorer friends, or should nursing be built up on a self-supporting basis, so that all draw together for a common purpose and have equal responsibilities and equal rights? Few would dispute that the latter course is the ideal to keep before the community, but it would appear from an examination of the facts that we are yet a long way from attaining that ideal, and that the short cuts to setting sick district nursing on an independent basis which are in practice in some localities are retarding rather than advancing the principle of justice in community life. In the first place it must be fully recognised that nursing is a costly luxury, out of the reach of people with small means, and generally, it may be said, of wage earners. The cost of district nursing, unless it be a wide organisation enabling the nursing to be done without waste of labour, may be estimated at from 1s. to 1s. 6d. a visit, and at an average of about 10s. a case. Under the Holt Ockley system of resident nurses the cost is seldom much less than £2 a case. An attempt is frequently made to assess the amount which each family ought to pay for blessings common to all. For this purpose the residents in a parish are divided into four classes—labourers, artisans, small shopkeepers and farmers, large farmers and professional men. Outside this list stands the landowner or man of independent means. Sometimes the division is effected on the rateable value of the house occupied. But in either case the individual is told that his contribution is to be 2s., 3s., 5s., or 10s., as the case may be, with a tacit understanding that the deficit must be made up out of the uncovenanted mercies of the rich. The system has several drawbacks. In the first place it is slightly inquisitorial. Then it is manifestly unfair, for the people with large families cannot under any system of classification be differentiated from childless couples. Again, it is very cumbersome, for it involves the collection of very small sums, often reluctantly given, which make up but an inconsiderable fraction of the cost. It is highly inconvenient; for how is it possible for the district nurse to neglect the sick poor who will not pay for her services? Lastly, it lays down a basis of contribution incapable of logical justification. Considered as payment, 2s. a year is not adequate; considered as donation it is manifestly far too much. This will become apparent if we examine the income at the disposal of the average labourer and that of his "betters." The agricultural labourer with six children earning 18s. a week is a fairly prosperous specimen of his class. Divide his income among the members of the family and the result is 2s. 4d. a week each. On this, house rent, food, clothing, medical relief, means of locomotion and recreations have to be provided for each person. It is done, often extremely well, but if the mother of the family should tell the friendly visitor anxious for their welfare that they cannot afford to pay for a trained nurse, is it remarkable? Contrast the income of this class, who are expected to contribute on the basis of 2s. a year to the fund, with the income of the small professional man who helps to organise the work. A man and his wife with private means of £400 a year are poor. With two children and £600 a year they are still poor. A lady living alone on £200 a year is very poor. Yet in all three cases the available weekly income for each member of the family is £3 16s. 11d., as contrasted with the labourer's 2s. 4d., and the extent of their contribution should logically be thirty-three times as much. Divide the half-guinea commonly expected for the nursing fund from gentle-folk with small means by thirty-three and the equivalent is seen to be no more than 3¾d. The basis of contribution breaks down in theory and in practice. In practice it gives an immense amount of trouble, and does not begin to make both ends meet.

But would you pauperise the labourers, it is asked with an anxiety which leaves altogether out of the question the true meaning of the word pauperise? A pauper is one who is supported by rates compulsorily levied on other members of the community. What has pauperism to do with a free gift generously bestowed on struggling mothers, on indigent old people, and ailing babies, by neighbours less heavily weighted down by the burden of poverty and hard work? "It blesseth him that gives and him that takes. . . ." Regarded in one aspect it is a luxury, for which the poor, even the struggling farmer or shopkeeper, the straitened parson or doctor with big families and depleted purses are quite unable to pay. Regarded from another point of view, it is an educational force of the highest social importance, which the ignorant and the neglected among the community do not yet appreciate, and which must be very gently and tactfully introduced into rural life if it is to do its perfect work. It is usual to say lightly no one is so poor that they cannot provide 2d. a month. But multiply this sum by thirty-three to make it answer to a small independent income, and its purchasing power to the labourer is revealed as 5s. 6d., and which of us would look amiably at the kind lady who called every month for this sum? There is perhaps no other free gift which we may press upon our neighbours with such confidence in doing them no mischief as that of skilled nursing in their hours of need.

Under wise management the poor will do their share. They will pay most thankfully for midwifery. They will contribute in most liberal and unexpected ways to the common fund of their own free will, and if the contributions be invited in some manner congenial to their incomes, through the institution of "pound day" or a local rummage sale, they will show splendid enthusiasm in making the function a success. But let us free ourselves from the pretence that district nurses can ever be made self-supporting by enforced contributions.

